# The prevalence of anxiety and its key influencing factors among the elderly in China

**DOI:** 10.3389/fpsyt.2023.1038049

**Published:** 2023-02-02

**Authors:** Yixuan Liu, Yanling Xu, Xinyan Yang, Guomei Miao, Yinghui Wu, Shujuan Yang

**Affiliations:** Department of Social Medicine and Health Management, School of Public Health, Jilin University, Changchun, China

**Keywords:** anxiety, influencing factors, random forest, mental health, aging

## Abstract

**Introduction:**

With the rapid aging population, the mental health of older adults is paid more and more attention. Anxiety is a common mental health illness in older adults. Therefore, the study aimed to explore the current situation of anxiety and its factors among the elderly in China.

**Methods:**

Based on the data from 2018 Chinese Longitudinal Healthy Longevity Survey (CLHLS), a total of 10,982 respondents aged 60 and above were selected. Generalized Anxiety Disorder (GAD-7) scale was used to assess the anxiety. Univariate and multivariate analysis were used to analyze the influencing factors of anxiety. Random forest was established to rank the importance of each influencing factors.

**Results:**

The results showed that the prevalence of anxiety among the elderly was 11.24%. Anxiety was mainly associated with 14 factors from five aspects: sociodemographic characteristics, health status, psychological state, social trust and social participation, among which loneliness related to psychological status was the most important factor.

**Discussion:**

The revelation of this study is that the present situation of anxiety among the elderly cannot be ignored, and it is necessary to take measures to prevent and control it from many aspects.

## 1. Introduction

In recent years, many countries around the world have experienced a rapid aging population, which has led to growing concern about the public health issues of elderly individuals, both physical and mental health. Similarly, as a country where the elderly comprise approximately 13.5% of its total population (1), China is currently facing the unprecedented challenges brought about by aging.

The pressure of the aging population includes a crushing global burden of disease and a greater demand for health services. In terms of mental health, a large number of studies have shown that more than 20% of elderly individuals suffer from different mental disorders, among which anxiety is one of the most common (2). As the sixth leading cause of disability worldwide (3), anxiety is a negative psychological emotion related to pain and mental discomfort (4). Studies have shown that 15–52% of older adults are affected by anxiety (5). Richardson et al. (6) found that more than 27% of elderly adults experienced anxiety symptoms, which significantly affected their mental health. Maatouk Imad et al. (7) analyzed data of the ESTHER-cohort study and found that the prevalence of anxiety among older adults form southwest Germany was 13.9%. In China, anxiety disorders were the most common class of disorders, with the lifetime prevalence of 7.6% (8). A cross-sectional study conducted among older adults in south China reported the prevalence of anxiety as 7.9% (9). A survey included 4,103 community-dwellings adults aged 60 and older from the West China showed that anxiety were prevalent in 20.8% of all participants (10). Although, the difference in the prevalence of anxiety might be attributed to different time-frame, research designs and studied population (limited by age, region, etc.). To be sure, due to population aging, especially in China, the absolute number of older people suffering from anxiety is increasing, which requires constant attention (11).

Compared with young people, the elderly with anxiety have difficulty paying attention, feel dizzy or faint, and experience nausea or diarrhea when worried, which can ultimately make them face debilitating pain (12). Previous research has indicated that elderly adults may experience decreased self-efficacy because of changes in their daily life and the environment (13). Furthermore, anxiety among the elderly is associated with numerous chronic physical illnesses and other negative emotions (14), such as pain and loneliness. Some studies of older people reported that the risk factors for anxiety involve being female, high neuroticism, hearing/visual impairments, lack of social participation and experiencing negative events (15, 16). By studying the relationship between anxiety and insomnia, Joshua et al. (17) found that anxiety and insomnia may share a common vulnerability to negative emotions, and they may sometimes be risk factors for each other. In summary, there are many factors linked with anxiety in elderly individuals, but the relative importance of these factors is still poorly understood and worthy of further in-depth study.

Anxiety is ubiquitous in elderly individuals, from the perspective of population aging, the pressure caused by anxiety includes the increasing global burden of disease, a greater needs for health services and increased mortality (5, 18, 19). For individuals, the negative outcomes of anxiety include diminished quality of life and well-being, disability, and even more severe physical and mental disorders (20). To prevent anxiety among the elderly, as well as provide meaningful support for subsequent therapeutic interventions, it is necessary to investigate and assess the contributing factors of anxiety adequately.

Most of the existing studies about anxiety among Chinese are based on specific populations [such as children and adolescents (21), some occupational groups (22), or patients with certain diseases (23)], medium sample size (10) (less than 10,000 respondents) and limited sample area (9) (studies are usually conducted in several cities or regions). The research method tends to be traditional logistic regression analysis. Few current studies have delved into anxiety among older adults using nationally representative data. As a machine learning algorithm, random forest is an excellent tool for learning feature representations because of its powerful classification ability and easily interpretable learning mechanism. In recent years, random forest has been widely used in the field of medicine to diagnose and classify diseases (24), predict clinical outcomes (25), and estimate the importance of exposure to pathogenic factors (26). Therefore, this study aimed to understand the prevalence of anxiety among the elderly in China through nationally representative data. Meanwhile, the study also analyzed the factors that may be related to anxiety from multiple perspectives, and measured the importance of these factors by random forest. This can provided an extremely significant reference for relieving anxiety symptoms and promoting overall mental health among elderly individuals.

## 2. Materials and methods

### 2.1. Study population

The data of this study came from Chinese Longitudinal Healthy Longevity Survey (CLHLS), which is organized by the Center for Healthy Aging and Development Studies (CHADS) of Peking University. Its collection began in 1998 and was followed up in 2000, 2002, 2005, 2008/2009, 2011/2012, 2014, and 2018, covering basic demographic characteristics, social, economic and health aspects. The sampling design of CLHLS adopted a multi-stage disproportionate and targeted random sampling method which makes the sample data representative and reliable (27). The data is nationally representative and has great value in theoretical study. The CLHLS study has got the approval of the Biomedical Ethics Committee of Peking University (IRB00001052-13074). Based on the 2018 CLHLS, the study selected the people aged 60 and above as respondents. We removed samples with answers like “I don’t know,” “I refuse to answer,” “not applicable,” and missing values for key variables. Finally, a total of 10,982 participants were included in the study for analysis.

### 2.2. Dependent variable

Anxiety was used as the dependent variable in this study. Participants’ anxiety was assessed by the 7-item Generalized Anxiety Disorder (GAD-7) scale translated into Chinese language (28). The scale has been proved to be applicable to Chinese older adults (29, 30). GAD-7 is composed of 7 items, including “feeling uneasy, worried and annoyed,” “can’t stop or can’t control worry,” “is worried too much about all kinds of things,” “is very nervous and it is difficult to relax,” “is very anxious, so you can’t sit still,” “becomes easy to get annoyed or easily irritated,” “feels like something terrible happens.” The options vary from 0 to 3 for each question (0 = never, 1 = for several days, 2 = more than half of days, and 3 = almost every day). The total score ranges from 0 to 21, with higher scores indicating more severe levels of anxiety among the elderly. A score greater than 4 was considered significant anxiety symptoms (31). The Cronbach’s alpha coefficient in this study was 0.914.

### 2.3. Independent variable

In this study, we classified the factors that might be associated with anxiety into 7 aspects: sociodemographic characteristics, lifestyle, health status, psychological state, social trust, social support and social participation. Specifically, the sociodemographic characteristics were as follows: age (0 = 81∼, 1 = 60∼80), gender (0 = male, 1 = female), occupation (0 = agriculture, 1 = non-agricultural), the area of residence (0 = rural, 1 = urban), and self-rated economic status(SRE) (1 = very rich to 5 = very poor). Marital status was coded into the following two categories: 0 = not married (never married, divorced or widowed), 1 = married (married or cohabitating). The form of residence was categorized as “non-alone” (with household member(s) or in an institution) and “alone.” Self-rated quality of life (SRL) and self-rated sleep quality (SRS) were recorded on a five-point scale as follows: 1 = very good; 2 = good; 3 = so-so; 4 = bad; and 5 = very bad.

Lifestyle consisted of current smoking status (0 = non-smoker, 1 = smoker), current drinking status (0 = non-drinker, 1 = current drinker), and current exercise or not (0 = no, 1 = yes).

Health status was assessed through seven areas: (1) Self-rated health status (SRH). Self-rated health status was assessed on a five-point scale: 1 = very good; 2 = good; 3 = so-so; 4 = bad; 5 = very bad. (2) Two-week morbidity. Two-week morbidity was evaluated with the following question: “Have you been feeling not-well within the past 2 weeks?” (3) Number of chronic disease. Total number of chronic diseases was grouped as 0, 1 and ≥2 (32). (4) Sensory impairment was identified including hearing and visual impairment. Hearing impairment referred to both complete and partial loss of the ability to hear. In the questionnaire, the interviewer assessed the hearing function of the respondents by asking them if they have difficulty listening. To assess visual impairment, the interviewer shone a flashlight at the circle on the chart and asked participants if they could see the circle and distinguish the direction of the break in the circle without glasses. There were four options: ➀ can see and distinguish; ➁ can see only; ➂ can’t see; ➃ blind. If respondents chose options 2–4, they were classified as visual impairment. (5) Obesity. According to previous studies and the constitution of Chinese people, waist circumference > 85 cm for males and >80 cm for females was considered as central obesity (33). (6) Activity of daily living (ADL) disability. ADL disability was measured by Katz Index (34), which includes six items: bathing, dressing, toileting, indoor transferring, feeding, control of defecation and urination. The answer of participants for each item was divided into three levels: ➀ without assistance; ➁ one part assistance; ➂ more than one part assistance. Participants who chose either ➁ or ➂ were considered ADL disability. As long as one of the six items was counted as disabled, they were assessed as having ADL disability. (7) Falls. Falls were evaluated through a single question: “Have you fallen in the past year?” The option was “Yes” or “No.”

In terms of psychological state, we applied a question to measure loneliness: “Do you feel lonely?” The scores ranged from 1 to 5, which meant that the lower the scores were, the greater the loneliness. Similarly, social trust was also measured by a five-point question: “Do you often feel that people around you are not trustworthy?” Higher scores indicated better trust in society.

Social participation refers to individuals’ reasonable participation in social activities (35). According to previous studies (30), social participation among the elderly was measured by group activities and self-recreation activities. Group activities were indicated by the following three aspects: outdoor activities (Tai Ji, square dance, visit and interact with friends, other outdoor activities), playing cards or mah-jong and some social activities. Self-recreation activities included garden work, reading newspapers/books, raising domestic animals/pets, watching TV or listening to the radio. The answers were recorded as 1 (almost every day) to 5 (never). We rated 1 to 3 as participating (Yes) and 4 to 5 as little or never participating (No). People who participated in any activity could be regarded as participating in group or self-recreation activities. Social support is divided into formal social support and informal social support. Formal social support refers to social security and social services provided by government agencies, enterprises and communities (36). Informal social support is defined as material support and non-material support provided by family, spouse and friends (37). In this study, social security was evaluated as whether the respondents had medical security (basic medical insurance and commercial medical insurance) and endowment security (including a pension and endowment insurance). We denoted social services using 9 items of services available for the elderly provided by the community: personal care services, home visit services, psychological consulting services, daily shopping services, social and recreation services, legal aid services, healthcare education service, neighborhood-relation services, and other social services. Respondents who answered that their community had one or more of these services were defined as “yes,” otherwise, they were defined as “no.” The study assessed informal social support using intergenerational material and non-material support for the elderly from children and grandchildren. Intergenerational material support was measured as whether respondents had received cash or in-kind contributions from their children or grandchildren in the past year (0 = no, 1 = yes). Intergenerational non-material support was measured by whether the respondents’ children or grandchildren visited or contacted them regularly and provided daily care when the respondents were unwell or ill (0 = no, 1 = yes).

### 2.4. Random forest

Random Forest is a machine learning method based on a classification tree, which can deal with both classification and regression analysis (38). The basic principle is that the bootstrap repeated sampling method is adopted to randomly extract some observed values of dependent variables from the dataset and select a specified number of variables among independent variables to determine the nodes of the classification tree. Random forest is an ensemble of hundreds or thousands of classification or regression trees that are created from a random selection of samples derived from the training data (39). The out-of-bag (OOB) proportion of the data left over during tree construction is used as validation data to calculate classification errors. The OOB method can be used to measure the importance of each independent variable. The values of each variable in the OOB sample were randomly arranged in turn, and the OOB error was calculated. If the OOB errors increase as the permutation value increases, it indicates that the variable is important. The greater the change is, the more important the variable is to the prediction of the dependent variable (38). In the aspect of evaluating the importance of variables, random forest algorithm estimates the error of the model through the OOB error (unbiased estimation of the prediction error), and the model has strong generalization ability, which makes its prediction results better than other machine algorithms such as back propagation neural networks (40).

### 2.5. Statistics analysis

IBM SPSS Statistics version 24 and RStudio were used for data analysis. First of all, descriptive analysis was performed on the samples. Chi-square test was used for univariable analysis. Secondly, the factors with statistical significance in univariate analysis were included in binary logistic regression to explore the influencing factors of anxiety in the elderly. In addition, RStudio was used to establish the random forest to evaluate and rank the importance of the influencing factors of anxiety. The level of statistical significance was *P* < 0.05(two-sided).

## 3. Results

### 3.1. Description of the sample

As shown in Table 1, a total of 10,982 respondents were included in this study, among which 1,234 had anxiety symptoms, accounting for 11.24%. In terms of age, 43.1% of the respondents were aged 60–80. The gender ratio was relatively even, with 46.3% males and 53.7% females. More than 70% of the respondents lived in rural areas (77.1%) and worked in agriculture (52.1%) and 47.1% were married. The overwhelming majority of the respondents were “non-alone” (83.3%), meaning they lived with family member(s) or in an institution. Seventy percent of the respondents believed their economic status were at the middle level of society, 70.4% were satisfied with their quality of life, and more than half slept well (52.7%). In terms of lifestyle, a minority of respondents were currently smoking (16.1%) or drinking (15.7%), and 66.4% had the habit of exercise. From the perspective of health status, 48% of the respondents rated their health as “good” or “very good.” In terms of psychological state, 25.4% of the respondents had a significant sense of loneliness, and most of them believed that people around them were trustworthy (74.2%). A number of respondents were involved in some group (66%) or self-recreation activities (78.4%). In addition, various social security and social services provided by the government, enterprises and communities basically covered more than half of the respondents, and most of them also received material and non-material support from their children or grandchildren, accounting for 76.8 and 96.3%, respectively.

**TABLE 1:** The demographic characteristics distribution of anxiety among the elderly and univariate analysis.

Variables	Total	Normal	Anxiety	χ^2^	*P-*value
	**(*N* = 10982)**	**(*N* = 9478)**	**(*N* = 1234)**		
Age				9.958	0.002
60∼80	4737 (43.1)	4153 (42.6)	584 (47.3)		
81∼	6245 (56.9)	5595 (57.4)	650 (52.7)		
Gender				68.295	<0.001
Male	5085 (46.3)	4650 (47.7)	435 (35.3)		
Female	5897 (53.7)	5098 (52.3)	799 (64.7)		
Marital status				6.374	0.012
Not married	5814 (52.9)	5119 (52.5)	695 (56.3)		
Married	5168 (47.1)	4629 (47.5)	539 (43.7)		
Occupation				22.857	<0.001
Agriculture	5722 (52.1)	5000 (51.3)	722 (58.5)		
Non-agricultural	5260 (47.9)	4748 (48.7)	512 (41.5)		
Area of residence				19.315	<0.001
Rural	8462 (77.1)	7450 (76.4)	1012 (82.0)		
Urban	2520 (22.9)	2298 (23.6)	222 (18.0)		
Form of residence				10.715	0.001
Non-alone	9152 (83.3)	8164 (83.8)	988 (80.1)		
Alone	1830 (16.7)	1584 (16.2)	246 (19.9)		
SRE				279.388	<0.001
Very rich	306 (2.8)	294 (3.0)	12 (1.0)		
Rich	1923 (17.5)	1803 (18.5)	120 (9.7)		
So-so	7685 (70.0)	6852 (70.3)	833 (67.5)		
Poor	954 (8.7)	721 (7.4)	233 (18.9)		
Very poor	114 (1.0)	78 (0.8)	36 (2.9)		
SRL				389.035	<0.001
Very good	2519 (22.9)	2364 (24.3)	155 (12.6)		
Good	5215 (47.5)	4734 (48.6)	481 (39.0)		
So-so	2936 (26.7)	2457 (25.2)	479 (38.8)		
Bad	275 (2.5)	171 (1.8)	104 (8.4)		
Very bad	37 (0.3)	22 (0.2)	15 (1.2)		
SRS				709.507	<0.001
Very good	1705 (15.5)	1615 (16.6)	90 (7.3)		
Good	4084 (37.2)	3854 (39.5)	230 (18.6)		
So-so	3533 (32.2)	3093 (31.7)	440 (35.7)		
Bad	1409 (12.8)	1025 (10.5)	384 (31.1)		
Very bad	251 (2.3)	161 (1.7)	90 (7.3)		
Smoke				11.790	0.001
Non-smoker	9213 (83.9)	8136 (83.5)	1077 (87.3)		
Smoker	1769 (16.1)	1612 (16.5)	157 (12.7)		
Drink				19.759	<0.001
Non-drinker	9260 (84.3)	8166 (83.8)	1094 (88.7)		
Drinker	1722 (15.7)	1582 (16.2)	140 (11.3)		
Exercise				14.656	<0.001
No	7290 (66.4)	6411 (65.8)	879 (71.2)		
Yes	3692 (33.6)	3337 (34.2)	355 (28.8)		
SRH				602.566	<0.001
Very good	1304 (11.9)	1257 (12.9)	47 (3.8)		
Good	3961 (36.1)	3705 (38.0)	256 (20.7)		
So-so	4274 (38.9)	3743 (38.4)	531 (43.0)		
Bad	1324 (12.1)	977 (10.0)	347 (28.1)		
Very bad	119 (1.1)	66 (0.7)	53 (4.3)		
Two-week morbidity				362.657	<0.001
No	9270 (84.4)	8457 (86.8)	813 (65.9)		
Yes	1712 (15.6)	1291 (13.2)	421 (34.1)		
Number of chronic disease				52.592	<0.001
0	3139 (28.6)	2867 (29.4)	272 (22.0)		
1	3492 (31.8)	3131 (32.1)	361 (29.3)		
≥2	4351 (39.6)	3750 (38.5)	601 (48.7)		
Hearing impairment				27.210	<0.001
No	7268 (66.2)	6533 (67.0)	735 (59.6)		
Yes	3714 (33.8)	3215 (33.0)	499 (40.4)		
Visual impairment				58.215	<0.001
No	7752 (70.6)	6996 (71.8)	756 (61.3)		
Yes	3230 (29.4)	2752 (28.2)	478 (38.7)		
Obesity				6.646	0.010
No	4179 (38.1)	3668 (37.6)	511 (41.4)		
Yes	6803 (61.9)	6080 (62.4)	723 (58.6)		
ADL disability				0.874	0.350
No	284 (2.6)	257 (2.6)	27 (2.2)		
Yes	10698 (97.4)	9491 (97.4)	1207 (97.8)		
Fall				128.247	<0.001
No	8645 (78.7)	7827 (80.3)	818 (66.3)		
Yes	2337 (21.3)	1921 (19.7)	416 (33.7)		
Loneliness				995.308	<0.001
Always	213 (1.9)	118 (1.2)	95 (7.7)		
Often	502 (4.6)	295 (3.0)	207 (16.8)		
Sometimes	2078 (18.9)	1703 (17.5)	375 (30.4)		
Seldom	3761 (34.2)	3408 (35.0)	353 (28.6)		
Rarely or never	4428 (40.3)	4224 (43.3)	204 (16.5)		
Social trust				408.048	<0.001
Very bad	734 (6.7)	3453 (35.4)	187 (15.2)		
Bad	849 (7.7)	4014 (41.2)	502 (40.7)		
So-so	1243 (11.3)	928 (9.5)	315 (25.5)		
Good	4516 (41.1)	709 (7.3)	140 (11.3)		
Very good	3640 (33.1)	644 (6.6)	90 (7.3)		
Group activities				41.755	<0.001
No	3735 (34.0)	3214 (33.0)	521 (42.2)		
Yes	7247 (66.0)	6534 (67.0)	713 (57.8)		
Self-recreation activities				41.837	<0.001
No	2375 (21.6)	2020 (20.7)	355 (28.8)		
Yes	8607 (78.4)	7728 (79.3)	879 (71.2)		
Medical security				0.043	0.836
No	1209 (11.0)	1071 (11.0)	138 (11.2)		
Yes	9773 (89.0)	8677 (89.0)	1096 (88.8)		
Endowment security				<0.001	0.996
No	4931 (44.9)	4377 (44.9)	554 (44.9)		
Yes	6051 (55.1)	5371 (55.1)	680 (55.1)		
Social services				1.292	0.256
No	3995 (36.4)	3528 (36.2)	467 (37.8)		
Yes	6987 (63.6)	6220 (63.8)	767 (62.2)		
Intergenerational material support				5.206	0.023
No	2544 (23.2)	2290 (23.5)	254 (20.6)		
Yes	8438 (76.8)	7458 (76.5)	980 (79.4)		
Intergenerational non-material support				17.271	<0.001
No	402 (3.7)	331 (3.4)	71 (5.8)		
Yes	10580 (96.3)	9417 (96.6)	1163 (94.2)		

SRE, self-rated economic status; SRL, self-rated quality of life; SRS, self-rated sleep quality; SRH, self-rated health status; ADL disability, activity of daily living disability.

This study found that the prevalence of anxiety was 11.24%. Compared with participants without anxiety, participants with anxiety were more likely to be older, female, not married, work in agriculture and live in rural areas. At the same time, the distribution of anxiety among the elderly with different characteristics was obtained by univariate analysis, which indicated that all sociodemographic factors and the variables included in life style, psychological state, social trust and social participation were significantly related to anxiety (*p* < 0.05). In terms of health status, except for ADL disability, the other six factors were significantly related to anxiety (*p* < 0.05). Regarding social support, only informal social support including intergenerational material and non-material support proved to be linked with anxiety (*p* < 0.05). Table 1 provides detailed information about the participants’ characteristics in this study.

### 3.2. Results of binary logistics regression

We further explored the influencing factors of anxiety among the elderly through binary logistics regression. The statistical results were shown in Table 2. According to the odds ratio, the elderly aged 60 to 80 had higher rates of anxiety than those aged 81 and over (OR = 1.621; 95% CI: 1.375, 1.911). Females were more likely to suffer from anxiety than males (OR = 1.503; 95% CI: 1.284, 1.759). People who were married had higher rates of anxiety than those who were not married (OR = 1.309; 95% CI: 1.104, 1.551). The elderly living in urban areas had a lower risk of anxiety than those living in rural areas (OR = 0.784; 95% CI: 0.644, 0.953). The respondents who rated themselves with a poor quality of sleep and life and in poor economic and health status had a significantly higher proportion with anxiety. Two-week morbidity (OR = 1.798, 95% CI: 1.534, 2.108) and falls (OR = 1.383, 95% CI: 1.191, 1.606) were also associated with anxiety. A lower level of loneliness (OR = 0.528, 95% CI: 0.494, 0.565) and a good sense of social trust (OR = 0.791, 95% CI: 0.748, 0.836) appeared to produce a certain protective effect against anxiety. People with hearing impairment were more likely to be anxious than those without hearing impairment (OR = 1.200; 95% CI: 1.034, 1.394). People who participated in group activities had a lower risk of developing anxiety (OR = 0.767; 95% CI: 0.658, 0.894).

**TABLE 2:** Binary logistic regression analysis of anxiety in the elderly.

Variables		OR	95% CI	*P*-value
Age	81	1.000		
	60–80	1.621	(1.375, 1.911)	<0.001
Gender	Male	1.000		
	Female	1.503	(1.284, 1.759)	<0.001
Marital status	Not married	1.000		
	Married	1.309	(1.104, 1.551)	0.002
Occupation	Agriculture	1.000		
	Non-agricultural	0.929	(0.799, 1.081)	0.339
Area of residence	Rural	1.000		
	Urban	0.784	(0.644, 0.953)	0.015
Form of residence	Non-alone	1.000		
	Alone	0.847	(0.702, 1.021)	0.082
SRE		1.300	(1.158, 1.460)	<0.001
SRL		1.109	(1.008, 1.221)	0.034
SRS		1.558	(1.450, 1.673)	<0.001
Smoke	Non-smoker	1.000		
	Smoker	0.957	(0.772, 1.186)	0.690
Drink	Non-drinker	1.000		
	Drinker	1.038	(0.834, 1.291)	0.740
Exercise	No	1.000		
	Yes	1.161	(0.992, 1.358)	0.063
SRH		1.300	(1.185, 1.426)	<0.001
Two-week morbidity	No	1.000		
	Yes	1.798	(1.534, 2.108)	<0.001
Number of chronic disease	0	1.000		
	1	1.076	(0.897, 1.291)	0.431
	≥2	1.137	(0.950, 1.361)	0.162
Hearing impairment	No	1.000		
	Yes	1.200	(1.034, 1.394)	0.017
Visual impairment	No	1.000		
	Yes	1.136	(0.978, 1.319)	0.095
Obesity	No	1.000		
	Yes	0.891	(0.775, 1.024)	0.104
Fall	No	1.000		
	Yes	1.383	(1.191, 1.606)	<0.001
Loneliness		0.528	(0.494, 0.565)	<0.001
Social trust		0.791	(0.748, 0.836)	<0.001
Group activities	No	1.000		
	Yes	0.767	(0.658, 0.894)	0.001
Self-recreation activities	No	1.000		
	Yes	0.859	(0.725, 1.018)	0.079
Intergenerational material support	No	1.000		
	Yes	1.082	(0.917, 1.277)	0.351
Intergenerational non-material support	No	1.000		
	Yes	1.107	(0.804, 1.523)	0.534

SRE, self-rated economic status; SRL, self-rated quality of life; SRS, self-rated sleep quality; SRH, self-rated health status.

### 3.3. Results of random forest

**FIGURE 1:**
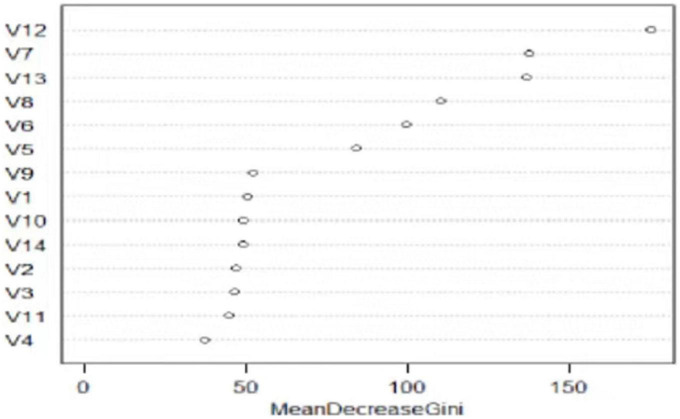
Ranking of the importance of different factors on anxiety among the elderly.

To further evaluate the importance of each influencing factor, we coded each factor as V1–V14 and then randomly selected 70% of the overall data as the training set and 30% as the test set to establish a random forest with parameters mtry 3 and ntree 500. The random forest algorithm measures the influence of each variable on the dependent variable by the variable importance score; that is, the greater the average reduction of the Gini index, the more important this variable is (41). Figure 1 and Table 3 showed the ranking results of influencing factors according to the mean reduction of Gini, which indicated that loneliness was the key factor related to anxiety of the elderly, and other influencing factors were ranked in order of importance as follows: SRS, social trust, SRH, SRL, SRE, 2-week morbidity, age, hearing impairment, group activities, gender, marital status, fall, area of residence.

**TABLE 3:** The rank of importance of the factors influencing anxiety among the elderly.

Rank	Variable	Mean Gini reduction
1	Loneliness (V12)	175.548
2	SRS (V7)	137.733
3	Social trust (V13)	136.723
4	SRH (V8)	110.279
5	SRL (V6)	99.876
6	SRE (V5)	84.213
7	Two-week morbidity (V9)	52.080
8	Age (V1)	50.350
9	Hearing impairment (V10)	49.369
10	Group activities (V14)	49.013
11	Gender (V2)	46.916
12	Marital status (V3)	46.481
13	Fall (V11)	44.825
14	Area of residence (V4)	37.230

SRE, self-rated economic status; SRL, self-rated quality of life; SRS, self-rated sleep quality; SRH, self-rated health status.

## 4. Discussion

Based on the data from 2018 Chinese Longitudinal Healthy Longevity Survey (CLHLS), this study explored the current situation of anxiety and its influencing factors among Chinese elderly people aged 60 and above. The study found that the prevalence of anxiety was 11.24%, which was consistent with previous studies for Chinese elderly adults (42). Furthermore, a random forest model was established to detect the importance of each factor more clearly. This study has reference significance for further understanding the current situation of anxiety among Chinese elderly individuals, and it also provides certain valuable enlightenment for the prevention of anxiety among the elderly. The crucial finding of this study showed that anxiety was mainly related to five aspects: sociodemographic characteristics, health status, psychological state, social trust and social participation. According to the random forest, loneliness had a great impact on anxiety, which meant that lonely older people were, more likely to become anxious. A longitudinal study among an older Irish population showed that loneliness was predictive of anxiety symptoms (43). As a specific psychological state, loneliness is defined as feelings and thoughts of isolation or disconnection from others (44). In China, the elderly traditionally live with family members, especially their spouses and adult children. However, with the development of urbanization, the traditional extended family is gradually disintegrating (45). Many children are leaving their parents and hometown to live in other cities or regions (46). Although many elderly people can take care of themselves, living apart from their adult children still causes them to feel deep loneliness. In addition, the changes of social roles make the quality of personal relationships among the elderly tend to decline (47). There is evidence that approximately one-third of older people suffer from loneliness at the end of their lives because of lacking family care and emotional comfort (48), while those aged 80 and over report feeling lonely all the time (49). Previous research has indicated that loneliness among older adults is a risk factor for poor mental and physical health outcomes such as anxiety (50). A strong sense of loneliness is generally associated with severe negative emotional states and negative psychological consequences, which can contribute to a wide range of physical and emotional health problems, as in the case of anxiety (51).

Self-rated sleep quality reflects the individual’s subjective perception of their daily sleep, and is also a general indicator widely used in assessment of sleep quality (52). Results from this study confirmed the effect of self-rated sleep quality on anxiety among the elderly. We found that older adults with poor quality of sleep were associated with higher levels of anxiety. Approximately 20−40% of older adults have complained of sleep problems or poor sleep quality (53). Evidence suggested that poor sleep quality negatively affected mental health and was a proximal contributor to individual mental disorders (54). Previous research also found that anxiety and sleep disturbances frequently go hand in hand (55). As a result, when the elderly feel like they are sleeping poorly, anxiety goes with them.

Another interesting finding of this study was that a good sense of social trust could effectively alleviate anxiety among the elderly. In essence, social trust is the embodiment of social relationship. It is a collection of various factors that shape interpersonal relationship, with universality and stability (56). Empirical studies have shown that social trust at the individual level is strongly associated with anxiety (57). Social trust is believed to relieve individual emotional responses to anxiety by increasing perceived or actual social support (58). In China, especially for elderly adults, social trust is mainly reflected in the family or a narrow circle of interpersonal relationships, such as trust in family members, neighbors, and friends (59). Elderly individuals with a good sense of social trust have the lower likelihood of reporting anxiety, because they can obtain more information and emotional support from the person around them to handle problems, which, in turn, reduces anxiety (60). Moreover, there is evidence that people with higher levels of social trust are more likely to report good health, which plays a protective role in reducing anxiety (61). To a certain extent, a higher level of social trust could protect the elderly from the adverse impact of negative emotion by acting as a “buffer” and preventing development of anxiety.

In addition to the above three most important factors related to anxiety among the elderly, we also found that the impact of health-related factors on anxiety was also noteworthy. The study showed that people with a lower level of self-rated health had a higher risk of anxiety. Similarly, 2-week morbidity, hearing impairment and falls may also cause anxiety. There is evidence that people may fear the potential consequences of poor health status, which would trigger the development of anxiety (62). As a means of measuring health status, SRH and 2-week morbidity are important predictors of anxiety. Hearing impairment and falls mean limited mobility and reduced independence. It is likely to erode older adults’ overall self-efficacy and aggravate their anxiety and uncertainty about life (63). In a word, all of these risk factors may increase the level of anxiety in older adults.

The study also proved that SRL and SRE were linked with anxiety among the elderly (ranked fourth and fifth in terms of importance). SRL and SRE can be regarded as people’s subjective evaluation of the quality of life and economic situation. The negative correlation between quality of life and anxiety has been well confirmed in many studies (64, 65). Previous studies have shown that lower quality of life was predictive of negative mental health outcomes (66). Likewise, it was also previously established that economic status has an impact on mental health through the material and psychosocial pathways (67). Higher economic status is associated with more affluent material conditions, which reduces the psychosocial stress caused by financial hardship (68). Compared with counterparts, people with low economic status are more likely to be affected by mental disorders such as anxiety due to their reduced self-esteem and increased life pressures (69). Past research has shown that the distinguishing characteristics among many patients diagnosed with anxiety include low income levels and economic status (70). Most elderly people have experienced changes in their social and life roles, which can affect their quality of life and financial situation. The elderly with lower life quality or economic status are more prone to mental health problems such as anxiety (71). Moreover, some sociodemographic characteristics (age, gender, marital status, and the area of residence) have also been found to have a certain influence on anxiety among the elderly. As people grow older and their life cycle draws to its close, some older adults find themselves unable to accept the death and illness of spouses or peers. They feel anxious about facing the increasing health limitations and their own impending death (72). In terms of gender, females have a higher prevalence of anxiety disorders than males, which can be attributed to the fact that females are more vulnerable to negative emotions (73). At the same time, there are the benefits of a supportive partner, which also have a positive effect on the elderly’s psychological wellbeing (74). The relatively higher socioeconomic status, accessible health-care services and adequate social basic resources for the Chinese urban elderly can make up for negative feelings and reduce anxiety compared to their rural counterparts (75). Regarding the role of social participation, participation in group activities could reduce the risk of anxiety. The reason is that participation in group social activities increases more opportunities of communication with others and emotional catharsis, which significantly reduces the feeling of anxiety among the elderly (29).

## 5. Limitations

The results of this study are based on cross-sectional data, and it is difficult to infer causality, which should be further verified by longitudinal studies. In addition, the study only explored the relationship between loneliness and anxiety. More measures of other psychological state should be introduced in the future. Finally, some variables were evaluated by single or self-reported items. The participants’ subjective awareness may affect the authenticity of the measurement results. In future research, enriching the diversity and objectivity of indicators will better guarantee the quality of the study.

## 6. Conclusion

In this study, we explored the prevalence of anxiety among elderly people aged 60 and above and analyzed the importance of various influencing factors of anxiety using random forest model. According to the results, loneliness, self-rated sleep quality and social trust had a great impact on anxiety, followed by self-rated health status, self-rated quality of life, self-rated economic status, 2-week morbidity, age, hearing impairment, group activities, gender, marital status, fall and area of residence. Based on this, we call on the whole society to pay attention to the mental health of the elderly. Relevant departments and policy-makers should formulate targeted actions to improve the health literacy of the elderly, such as carrying out health education, providing emotional care for lonely elderly group, encouraging the elderly to participate in social activities and enriching their interpersonal relationship, improving regional infrastructure and increasing social welfare and living standards of elderly individuals. These measures are meaningful to attenuate the impact of negative emotions such as anxiety and maintain the psychological health of the elderly.

## Data availability statement

Publicly available datasets were analyzed in this study. This data can be found here: https://opendata.pku.edu.cn/dataverse/CHADS.

## Ethics statement

The CLHLS has been approved by the research ethics committees of Peking University (IRB00001052-13074). Written informed consent was not required as per local legislation and institutional requirements.

## Author contributions

SY: conceptualization, validation, and writing — original draft. YL: conceptualization, data curation, formal analysis, and writing — review and editing. YX and XY: formal analysis and writing — review and editing. GM and YW: editing. All authors contributed to the article and approved the submitted version.
